# Implementation of System Operation Modes for Health Management and Failure Prognosis in Cyber-Physical Systems

**DOI:** 10.3390/s20082429

**Published:** 2020-04-24

**Authors:** Santiago Ruiz-Arenas, Zoltán Rusák, Ricardo Mejía-Gutiérrez, Imre Horváth

**Affiliations:** 1 Faculty of Industrial Design Engineering, Delft University of Technology, Landbergstraat 15, 2628 CE Delft, The Netherlands; z.rusak@tudelft.nl (Z.R.); i.horvath@tudelft.nl (I.H.); 2 Design Engineering Research Group (GRID), Universidad EAFIT, Carrera 49 N° 7 Sur-50, Medellín 050001, Colombia; rmejiag@eafit.edu.co

**Keywords:** cyber-physical systems, failure prognosis, health management, system operation modes, system maintenance, system reliability

## Abstract

Cyber-physical systems (CPSs) have sophisticated control mechanisms that help achieve optimal system operations and services. These mechanisms, imply considering multiple signal inputs in parallel, to timely respond to varying working conditions. Despite the advantages that control mechanisms convey, they bring new challenges in terms of failure prevention. The compensatory action the control exerts cause a fault masking effect, hampering fault diagnosis. Likewise, the multiple information inputs CPSs have to process can affect the timely system response to faults. This article proposes a failure prognosis method, which combines time series-based forecasting methods with statistically based classification techniques in order to investigate system degradation and failure forming on system levels. This method utilizes a new approach based on the concept of the system operation mode (SOM) that offers a novel perspective for health management that allows monitoring the system behavior, through the frequency and duration of SOMs. Validation of this method was conducted by systematically injecting faults in a cyber-physical greenhouse testbed. The obtained results demonstrate that the degradation and fault forming process can be monitored by analyzing the changes of the frequency and duration of SOMs. These indicators made possible to estimate the time to failure caused by various failures in the conducted experiments.

## 1. Introduction

The cyber-physical system (CPS) concept, describes a family of systems that tightly connect the physical world with the information (cyber) world and obtain control information directly from real life processes, very often in run time and in real time [1]. These types of systems allow sensing physical variables, taking decisions, and actuating in real-time in order to adapt themselves to the varying environmental conditions.

Currently available CPSs are equipped with self-tuning capabilities that provide sophisticated control mechanisms that help to achieve stable, and optimal system operation. It enables the system to modify its own set points by changing the operational intensity of the actuators, as well as their activation and deactivation times, in order to respond to external disturbances. A typical example is a cyber-physical greenhouse, aiming to keep the best environmental conditions for the crops. If there are changes on the surrounding environment (e.g., like temperature decrease), the CPS will modify its settings, by increasing the intensity of the heater (or by extending the time it last on), and closing the windows, in order to keep the desired temperature. These new settings determine a system operation mode.

The adaptation capability that CPSs present, facilitates the provisioning of a range of new services including:

- Autonomous and optimum control of complex infrastructures (such as nuclear plants, traffic systems and air control systems, among others),
- Monitoring of complex physical processes,
- Provisioning of critical services in geographically distributed environments.

Due to the criticality of the provisioned services, CPSs should assure a continuous and reliable operation, protecting human life, the surrounding environment and the involved economical assets. This situation strengthens the need of preventing failures, and conducting a suitable fault management whenever it is needed.

Current systems are equipped with online measurement tools that support decision making and adjust system operation in real time [2]. These types of mechanisms enable fault detection and location, as well as the modification of system operation for fault effects reduction. Nevertheless, the effectivity of these mechanisms can be affected by the large amount of data CPSs have to process in parallel (e.g., energy consumption, business objectives, time restrictions, deadlines and volume of work, among others). This overwhelming amount of data may lead to delays in fault detection due to saturation, jeopardizing system operation.

Health management and failure prognosis has emerged as an opportunity for assuring the reliable operation of CPSs. These methods enable the real-time measuring of system variables in order to determine the extent of deviation from the normal operative conditions [3]. It allows evaluating the symptoms associated to degradation before they reach the critical threshold when the system collapses, providing time for planning maintenance actions and assuring the completion of system tasks [4]. Time-to-failure (TTF), remaining useful life (RUL), future health and/or risk to operation [5] are widely used as indicators for evaluating system reliability.

Existing health management and failure prognosis methods do not manage to cope with the intensive interaction between CPSs and their surrounding environment. They often present some of the following limitations: (i) they implement important simplifications in order to model the behavior of systems and their aging processes, (ii) the adaptation mechanisms of CPSs lead to disturbances in system signals that can be misinterpreted as faults, (iii) some of the existing methods make assumptions about the deterioration process of systems, which do not apply in dynamic and changing systems such as CPSs, and/or v) they are underpinned on empirical or experts knowledge, that cannot be easily updated and is limited to situations previously experienced.

Besides of the already mentioned limitations, the changing operative conditions of CPSs constitute the main challenge for the existing failure prognosis and health management methods. Self-regulation capabilities enable CPSs to compensate early-phase fault effects, assuring the desired system performance. However, they also mask fault symptoms hindering fault detection and diagnosis [6]. This affects the way in which fault forming and wearing out processes are depicted on system signals, preventing signals to present fault-induced alterations or progressive deviations that could be used for failure prognosis. This calls for development of new failure descriptors that can be used for failure prognosis.

This article, proposes a novel health management and failure prognosis method that aims to overcome the aforementioned limitations. Unlike traditional approaches that are mostly focused on studying system signals, the proposed method studies frequency and duration changes of system operation modes to predict remaining useful life of small-scale, self-tuning CPSs. The proposed method presents an important advantage over the existing approaches. It is a non-dedicated fault and system degradation indicator that can be used in any kind of small scale CPS. The proposed method combines time-series based analysis, and linear discriminant analysis. Time-series based analysis was utilized for forecasting the future values of system operation mode frequency and duration. It was also used as input for the failure prognosis model. Linear discriminant analysis was used to determine the failure mode causing the degradation process.

This article is organized as follows: Section 2 explores the literature in order to analyze the currently existing failure prognosis approaches, eliciting their limitations and opportunities concerning self-tuning CPSs. In Section 3, the main terms and concepts underpinning the proposed method are presented. Section 4 introduces and explains in detail the proposed failure prognosis method. Section 5 presents the validation of the proposed method, including a description of the instrumented testbed, the conducted experiments and the obtained results. Section 6 analyzes and discusses the implications of the observed results, considering the main characteristics of self-tuning CPSs. Finally, Section 7 summarizes the main conclusions of this research.

## 2. Current State of Failure Prognosis Methods

Despite failure prognosis in CPSs is not widely covered in the literature, there are multiple approaches available for complex systems. When analyzing literature, it can be observed that these approaches can be split into those, which are focused on determining the expected life of systems based on static models, and those that estimate system reliability indicators in run-time (failure prognosis in running-time).

The implementation of static-models for failure prognosis are mainly based on statistical analyses that aim to evaluate the accumulated failure rate of a sample of components, in order to determine their average useful life. These methods are developed based on the assumption that the obtained time to failure records fit a particular statistical distribution [7], which can be used to represent the operational deterioration, aging and wearing processes that occur once the useful life of systems have started [8].

One of the most commonly used types of statistical distribution is Weibull. Weibull distribution allows representing the “decreasing, constant and increasing part of the bathtub curve” [8] that describes a regular system deterioration process, enabling prognostics. This simplification of the process enables getting an estimated TTF that is used as basis for maintenance tasks, while it enables getting profit from the multiple parameters that can be used in this kind of statistical distribution. Linear-regression models are also considered as statistics based approaches [9]. As Weibull distribution, these approaches also evaluate the accumulated failure rate of a sample of components of the same type in order to determine their expected life, if these have not been subjected to renewal or repair processes.

Although statistics based models are easy to interpret, not all systems present the same tub curve [9]. These methods are not able to handle intermittent faults, and manage external conditions that can affect the regular operation of the system [3]. It causes the failure of even the most rigorous testing to simulate all the real-life situations of the analyzed systems [10]. This is particularly critical in CPSs as the self-tuning behavior, which characterize these systems, leads to a modified regime of system operation, also affecting their wearing pattern and deterioration of components.

Time-series-based analysis can overcome the limitations of the statistical-based analysis. In these methods, changes of the failure indicator variable are explained based on past observations [11]. Unlike traditional statistical methods, they do not assume a statistical distribution for representing the deterioration process. These methods consider specific features of time series data such as seasonality, trends and data cycles as basis for forecasting, facilitating their use as dynamic models that can be upgraded in run-time. The most common methods in this category are ARMA, ARIMA and exponential smoothing [12]. Some implementations can be found in [13,14,15].

Although the time-series-based methods are suitable for failure prognosis, the self-tuning capabilities of CPSs represent a challenge for their implementation. The system operation mode (SOM) transitions triggered by self-tuning control mechanism of the system may influence the trends of signals that are associated to deterioration. Likewise, they also hamper the occurrence of seasonal and sequential patterns that are necessary information sources of failure prognosis. The lack of proper failure indicators in self tuning systems is a driver for exploring failure descriptors that characterize the deterioration pattern caused by progressive failures and enable time-series-based failure prognosis.

Data-driven methods can also be used for failure prognosis in run-time. It makes them suitable for conducting health management. Some of the most common methods reported in literature are the Wiener process, Gamma process and Markovian models.

Wiener process is a data-driven method that can represent non-monotonic degradation processes (i.e., processes depicted by functions whose slope varies between positive, zero and negative), in order to estimate the remaining useful life (RUL) of systems and their time life [16]. This method uses information coming from the current degradation data, while ignoring previous observations. As a matter of example, regarding the application of this method, [17] Implemented a multiphase Wiener degradation model for predicting the storage life of high-voltage-pulse capacitors; [18] tackled the wear problem of magnetic heads used in hard disk drives (HDDs), by implementing Wiener processes with measurement errors. [16] Combined a Wiener-process-based degradation model with a recursive filter algorithm in order to estimate the RUL of an inertial navigation system, and [19] implemented a Wiener process in order to predict the lifetime of LED devices. In this last article, the author argues that traditional methods cannot capture dynamic and random variation of the LEDs degradation process. Despite of its wide use, Wiener processes are not suitable for modeling monotonic deterioration processes, they assume that faults present time-homogeneous evolution (while these are generally time-heterogeneous) and they ignore the past observations that can be critical for determining the occurring failure mode.

Unlike the Wiener process, the Gamma process is used for modeling monotonic degradation processes where deterioration takes place gradually [20]. As for Wiener process, there are plenty of applications of this method reported in literature: [21] proposes a remaining useful lifetime estimation, by considering a simulated noisy observation set corresponding to a Gamma process with additive Gaussian noise; [22] utilized degradation data in order to simulate the degradation process of components by implementing Gamma process and [23] aimed to predict the residual useful life of a component, by implementing an adaptive gamma process. The author used a state space model for updating the parameters of the gamma model whenever a new observation was available. Gamma process presents important limitations too. Although the fault evolution pattern presented in regular systems is monotonic (in which degradation occurs only in one direction), it may not be relevant to CPSs. The self-tuning behavior of CPSs compensates for the effects of fault and deterioration, leading to a non-monotonic behavior. Moreover, the Gamma process assumes that deterioration will occur in a sequence of tiny positive increments [20], while fault evolution can present a strong trend characterized by stepped progressions.

Markovian models are, by far, one of the most popular methods for failure prognosis in run-time and health management. This method estimates the forthcoming system degradation based on the current degradation state, ignoring the past observations. One of its main characteristics is that Markovian models evaluate the transition probability among states in order to determine the future state of the system [3]. For instance, [24] modeled the degradation states of a system through a set of hidden Markov models, in order to estimate the remaining useful life and the risk of an imminent fault in the future; and [25] proposed a framework for multisensory equipment diagnosis and prognosis based on adaptive hidden semi-Markov model. In this last application, the hidden semi-Markov model was used to identify the hidden degradation state, and the transition probabilities among health states.

Limitations of Markovian models include the memoryless assumption that may cause neglecting relevant information for estimating the future system state, or for diagnosing the forming failure mode. Likewise, transition probabilities are set based on empirical knowledge or through an important number of samples that are difficult to obtain [20]. This last factor should not be unattended, as there are some failure modes that are not known a priori and, thus, their probability cannot be estimated in advance. This situation is particularly critical in CPSs, due to the already mentioned varying working conditions and the emergent faults they are prone to occur during operation.

The above-presented literature review cast light on some important research challenges concerning the development of failure prognosis methods for CPSs:

- The applicability of existing methods has several prerequisites for failure indicators (e.g., monotonic, time-homogeneous, tiny-positive increments, etc.) and/or prior knowledge of the impact of failure on system behavior. These prerequisites are not applicable for CPSs, as these systems present dynamic behavior and operate in environments subjected to high levels of uncertainty.
- The non-predictable degradation presented by CPSs make the application of predefined statistical distribution patterns unreliable for capturing the process.
- The varying working conditions that characterize CPSs demand application of failure prognosis models that can be updated in run-time.
- There is lack of failure prognosis methods that do not rely on prior knowledge regarding failure manifestations, failure probability, and preventive means.
- There are not “universal” indicators that can be used for forecasting multiple failure modes.
- Methods used for prognosis in other disciplines should also be studied in order to find new ways to study the failure forming process in CPSs.

In conclusion, any failure prognosis method addressed to CPSs should be executed in run-time (based on sensed system performance); it should not be based on assumptions concerning the degradation process, or to rely on a predefined degradation path; and it should overcome the masking effect caused by fault tolerance.

## 3. Background and Theory Underpinning the Proposal

### 3.1. System Operation Modes

CPSs should adapt themselves to changing operating conditions in order to assure optimal performance. For this purpose, CPSs adapt their operational behavior by autonomously changing their system settings in response to sensed disturbances. This self-tuning mechanism is typically operationalized by modifying the working parameters of system actuators, which in conjunction determine the system’s operation mode (SOM).

SOMs can be considered as a subset of the state concept, as they describe the situation of the system at a particular time t [26]. A system state is traditionally defined by a set of variables that in conjunction provides relevant information for characterizing system behavior. The set of possible states a system can take are determined by the state space of the system [27]. The approach proposed in this article for SOMs considers actuators settings (called from now on as component operation modes) as the variables used as input for characterizing system behavior. Based on this definition, it can be said that the SOM state space is determined by the potential combinations of component operation modes, which are determined in turn by system actuators.

For the sake of a formal treatment, SOM has been defined as a singular combination of operation modes (COM) of all components of the system in a particular time t. COMs are regarded as the component state at a time t. The actuators can obviously be in multiple various states during their operation. As the most basic ones, in our study we considered the active and inactive states. For instance, the states of an outflow valve in charge of irrigation in a greenhouse can be symbolically represented as $E_{S_{A}{}_{j}}=\left\{\mathrm{ValveClose},\;\mathrm{ValveOpen}\right\}$, where E denotes the set of COMs of a particular component, and $S_{A}{}_{j}$ indicates the signal coming from the actuator j (which is the outflow valve in the above example). COMs of all system actuators at a time t defines the SOM at a time t, so that:

(1) $$\varsigma_{d}=\left\{\zeta_{S_{A_{1}}}\left(t\right),\zeta_{S_{A_{2}}}\left(t\right),\zeta_{S_{A_{3}}}\left(t\right),\zeta_{S_{A_{4}}}\left(t\right)\right\}$$

where: $\varsigma_{d}$ denotes the system’s operation mode d, and $\zeta_{S_{A}{}_{j}}\left(t\right)$ denotes the component operation modes of (four) actuators, with j in [1,4].

This SOM concept aims to capture the dynamic and adaptive system behavior in failure analytics, as it allows representing system behavior based on the joint effect of the actuators’ states on system performance. It relies on analyzing changes of component operation modes, which normally leads to the new SOMs that enables failure prognosis regardless of changes in system configuration. This article will focus CPSs built from components with discrete two-state (binary) COMs, i.e., active and inactive states, as a first approach for exploring the applicability of SOMs in failure prognosis.

### 3.2. Frequency and Duration of SOMs

CPSs change their system operation modes in order to assure optimal system performance for every working condition. This adaptation process can be described by the following sequence of steps: (i) system sensor perceive changes on the surrounding environment or system performance, (ii) the control system evaluates these changes and activate specific COMs to respond to the new working condition and (iii) the new combination of COMs leads to a new SOM.

SOM changes occur every time there are variations on the working conditions. Under regular system operation, all systems tend to activate certain SOMs more frequently than others. These SOMs are usually activated with a similar frequency, in akin time windows. Likewise, SOMs are prone to present a similar duration time, every time they are activated. Based on this line of reasoning, it can be argued that system faults, or the occurrence of abnormal events affects the frequency of occurrence and duration time of SOMs, making possible their use as fault indicators. This phenomenon has already been reported in [28], where the concept of failure-induced operation modes (FIOM) was introduced. This concept states that certain faults may lead to the occurrence of new SOMs that are not common in fault-free operation.

In order to provide a formal definition, in this article, the term SOM’s frequency (*Fq*) will refer to the number of times a SOM is activated during a time period, while the term SOM’s duration (*D*) is the average time while a SOM is active.

## 4. Description of the Proposed Computational Forecasting Method

The proposed heath management and failure prognosis method evaluates variations in *Fq* and *D* in order to detect faults, and estimating the system’s time to failure. It is claimed that, a particular failure mode tends to affect the same set of variables every time it occurs [28]. As a consequence, the system manipulates the same set of actuators as compensation, leading to similar variations in *Fq* and *D*, whenever the failure mode is repeated. In contrast, a different failure mode affects a different set of variables, leading to different variations in *Fq* and *D*.

**Figure 1:**
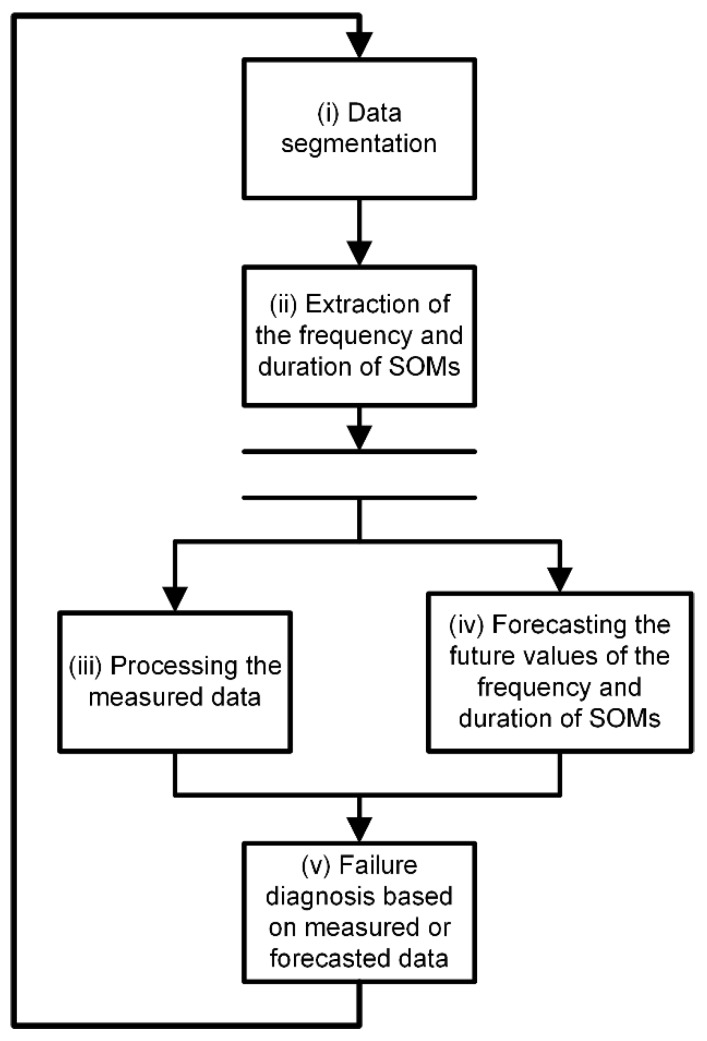
Procedure of the proposed computational failure forecasting method.

The proposed method includes five main stages of execution, namely: (i) data segmentation, (ii) extraction of the frequency and duration of SOMs, (iii) processing the measured data, (iv) forecasting the future values of the frequency and duration of SOMs and (v) failure diagnosis based on measured or forecasted data (Figure 1). These stages were explained in detail in the following paragraphs:

### 4.1. Data Segmentation

**Figure 2:**
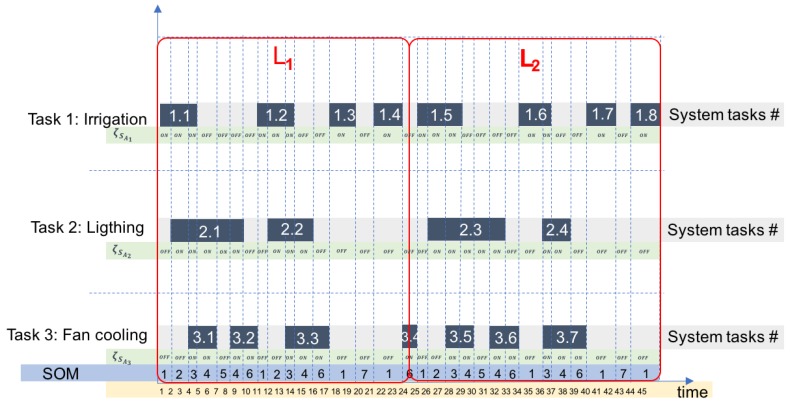
Tasks versus time diagram.

The frequency and duration of SOMs was estimated in fixed time-windows, in order to determine the regular number of occurrences of every SOM and their regular duration time in a standardized time length (L). This time length (L) should be set, according to: (i) main system tasks (processes), (ii) time required for tasks accomplishments and (iii) task sequences. A scheme that graphically represents system tasks versus their occurrence time is shown in Figure 2. This scheme depicts task sequences to determine if there are patterns in terms of duration time, parallel occurring tasks, sequence of occurrence, and time between task occurrences. It also allows observing the occurring SOMs and their sequences based on the deployment of the parallel system tasks. For the sake of clearness, we used the case of a cyber-physical greenhouse as example in Figure 2. Nevertheless, this approach could be used in any small-scale CPS.

The time length (L) was determined through an iterative process in which the size of L was systematically changed. In every iteration, multiple time-windows with the same L were sequentially arranged, in order to analyze if the key tasks present a similar number of occurrences. This iterative process was stopped whenever a convergence in the size of the different windows was achieved. It allowed for assigning a fix value to L for providing means to make comparisons between the number of occurrences and duration time of every SOM, from different data samples.

### 4.2. Extraction of the Frequency and Duration of SOMs

Once the standardized time length L has been set, the frequency and duration of every SOM in L should be estimated. This stage aims to get historical data of Fq and D for using them (in further steps) as input to derive the failure-prognosis model.

In this article, the “fault progress step” would be denoted as w, to refer to fault evolution at a given sampling rate. Every w contains data of SOM Fq and D during L that is derived by computing the number of SOM activations that occur within this time period and the time between activations, respectively. In every w the number of event occurrences of each SOM was counted and stored in a vector $F_{q_{\varsigma_{w}}}$. The duration time of every SOM was computed, averaged and stored in a vector $D_{\varsigma_{w}}$, where, ς denotes the analyzed SOM, and w the current fault progress step.

The results computed by this algorithm were stored in two matrices. The first one is the operation mode represented by a L×n matrix and denoted by OM (see Equation (2)). It is composed by status of all system actuators ($S_{A}$) at time *t* (being L the number of samples considered in w and n the number of actuators which are included in the analyzed system). In this matrix $S_{A}\;\left(t\right)=[S_{A_{1}}\left(t\right),\ldots ,\;S_{A_{n}}\left(t\right)]$, where $S_{A_{1}}\left(t\right)$ denotes the status of the actuator i at time *t*. In OM, every single row represents the SOM occurring at time *t*.

(2) $$OM_{L\times n}=\left[\begin{array}{c} \begin{array}{c} S_{A_{1}}\left(t=1\right),\ldots ,\;S_{A_{n}}\left(t=1\right)\\ S_{A_{1}}\left(t=2\right),\ldots ,\;S_{A_{n}}\left(t=2\right)\\ \vdots \end{array}\\ S_{A_{1}}\left(t=L\right),\ldots ,\;S_{A_{n}}\left(t=L\right) \end{array}\right]$$

The second matrix is a d×n matrix, denoted POM (see Equation (3)). It describes all potential SOMs $\varsigma_{j}$ that may appear in the system (considering all the possible combinations of COMs), being *d* the number of SOMs that the system may present. Every single row in POM was composed of a vector of length *n* like the one described in Equation (1).

(3) $$POM_{d\times n}=\left[\begin{array}{c} \varsigma_{1}\\ \vdots \\ \varsigma_{d} \end{array}\right]$$

**Figure 3:**
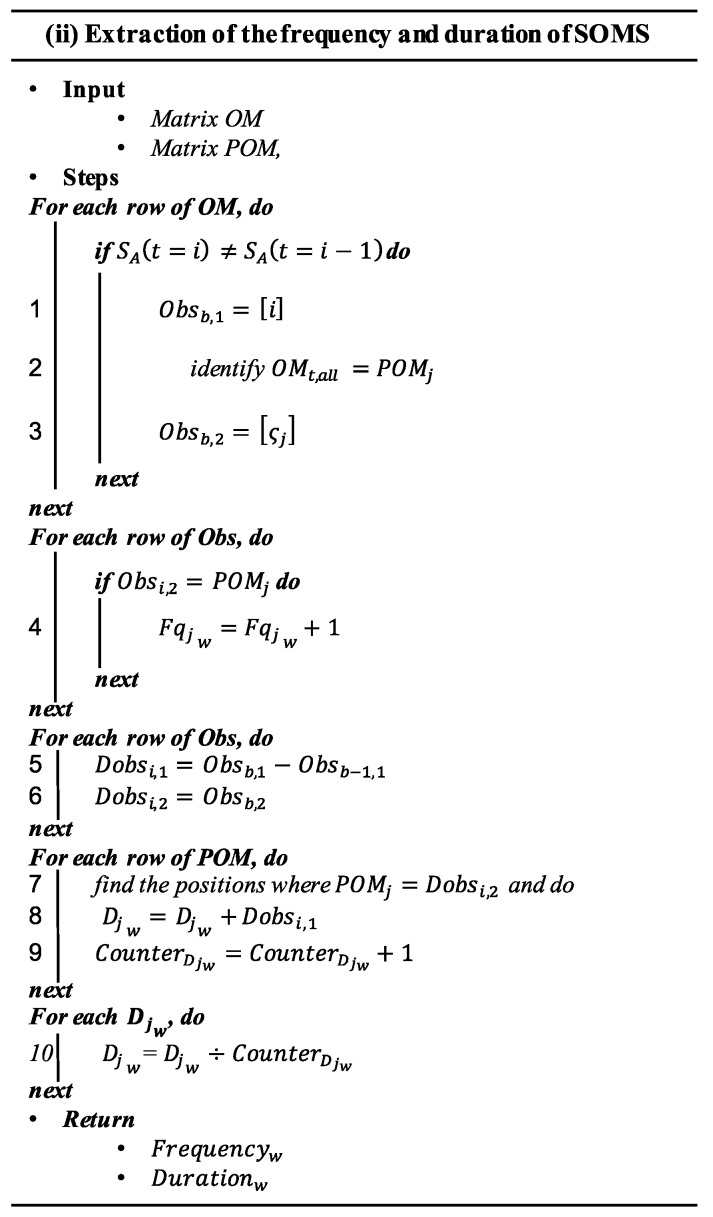
Pseudo-algorithm of step ii.

The detailed procedure is described in Figure 3, where the algorithm to extract the frequency and duration of SOMs, is presented.

In algorithm (i), $Obs_{b,1}$ denotes the vector that stores the time instants in which a new SOM is activated, as well as the occurring SOM ($\varsigma_{j}$) (with *b* = 1, …, c, where c is the total number of transitions observed); $Dobs_{i,1}$ stores the duration of every SOM, each time it occurs, and $Counter_{D_{j_{w}}}$ stores the number of times SOM $\varsigma_{j}$ occurred during the fault progress step w. This step returns $Frequency_{w}$ and $Duration_{w}$, which are expressed as:

(4) $$Frequency_{w}=\left[F_{q_{1_{w}}},F_{q_{2_{w}}},\ldots ,F_{q_{j_{w}}}\right]$$

(5) $$Duration_{w}=\left[D_{1_{w}},D_{2_{w}},\ldots ,D_{j_{w}}\right]$$

$F_{q_{j_{w}}}$ describes the observed frequency and $D_{j}._{w}$ the average duration of the jth SOM extracted at the time window *w*.

### 4.3. Processing the Measured Data

This step aims to eliminate the variations that may occur in the Fq and D data, due to minor fluctuations caused by varying working conditions. For this purpose, the Savitzky–Golay filter was proposed [29].

**Figure 4:**
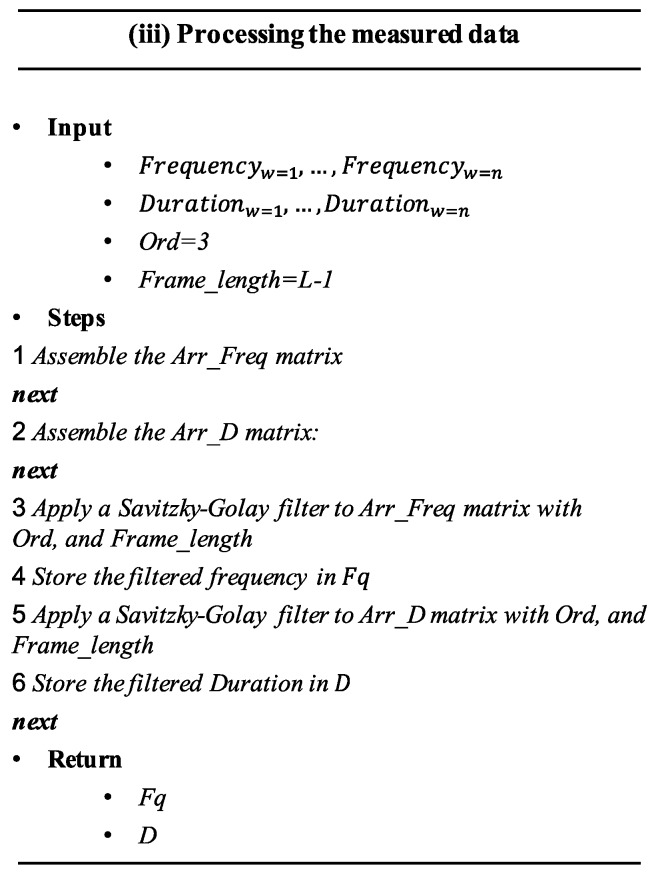
Pseudo-algorithm of step iii.

The algorithm (ii) related to this step (see Figure 4) uses $Frequency_{w}$ and $Duration_{w}$ vectors as input composed by $F_{q}$ and D data measured in consecutive fault progress steps; (*Ord* = 3), which denotes the polynomial order applied by the Savitzky–Golay filter, and Frame_length, which denotes the number of equally spaced data-points to be obtained after the application of the filter. Vectors corresponding to frequency are temporally put together in a n×L matrix, called Arr_Freq. Likewise, vectors corresponding to duration are temporally set in a n×L matrix, called Arr_D. Both matrices are expressed as

(6) $$Arr\_Freq_{n\times l}=\left[\begin{array}{c} Frequency_{1}\\ \vdots \\ Frequency_{n} \end{array}\right]$$

(7) $$\;\;\;\;\;\;\;\;\;\;\;Arr\_D_{n\times l}=\left[\begin{array}{c} Duration_{1}\\ \vdots \\ Duration_{n} \end{array}\right]$$

Arr_Freq and Arr_D were used as input for a Savitzky–Golay filter, whose results were stored in two n×L matrices, *Fq* and *D*. These matrices contain the frequency and duration data of all observed SOMs from different and consecutive fault progress steps. This step returns *Fq* and *D*.

### 4.4. Forecasting the Future Values of Frequency and Duration of SOMs

**Figure 5:**
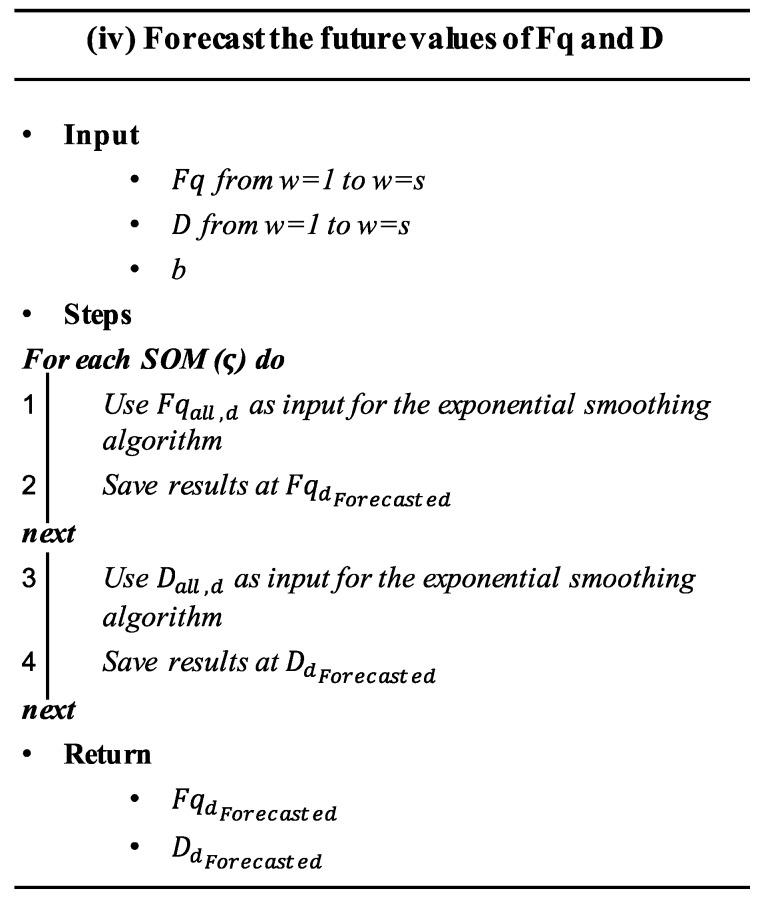
Pseudo-algorithm of step iv.

This step (detailed in algorithm (iii) shown in the Figure 5) aims at forecasting the forthcoming values of Fq and D based on their historic records. With this objective in mind, a time series-based forecasting model is proposed to extrapolate Fq and D of each SOM $\varsigma_{d}$ in a future time interval [t+1, …t+b], where *b* denotes the forecasting horizon. This process can be described as:

(8) $$F_{q_{d_{w+b}}}=f(F_{q_{d_{w}}},F_{q_{d_{w-1}}},\ldots ,F_{q_{d_{w-s}}})$$

(9) $$D_{d_{w+b}}=f(D_{d_{w}},D_{d_{w+1}},\ldots ,D_{d_{w-s}})$$

where s is the total number of fault progress steps considered in the estimation of $Fq_{w+b}$ and $D_{w+b}$. The term ‘fault progress step’ will be used in this article to denote the sampling considered for measuring the degradation process, or fault size throughout its forming process.

In this step, the *Fq* and *D* matrices obtained in step iii were used as input for the forecasting algorithm, as well as the forecasting horizon *b*. The extrapolated data of frequency and duration of SOMs were stored in $Fq_{d_{Forecasted}}$ and $D_{d_{Forecasted}}$ respectively, which are vectors of *b* elements.

### 4.5. Failure Diagnosis Based on Measured or Forecasted Data

This step can be conducted, either to diagnose actual failures or to analyze forming faults. Failure diagnosis is performed once a fault has reached its critical threshold by using measured data of Fq and D. This measured data was utilized as input for training the prognosis algorithm. In contrast, the failure prognosis aimed to detect faults before they reach their critical threshold w=O by using the forecasted data of Fq and D $Fq_{d_{Forecasted}}$ and $D_{d_{Forecasted}}$.

All the $Fq_{d_{Forecasted}}$ and $D_{d_{Forecasted}}$ vectors derived in step (iv) were put together into two new matrices denoted as $Fq^{\prime }$ and $D^{\prime },$ where $Fq^{\prime }$ contained all the forecasted data corresponding to frequency, and $D^{\prime }$ all the forecasted data corresponding to duration. Fq and D would continue to be used for referring to the observed data of frequency and duration respectively.

**Figure 6:**
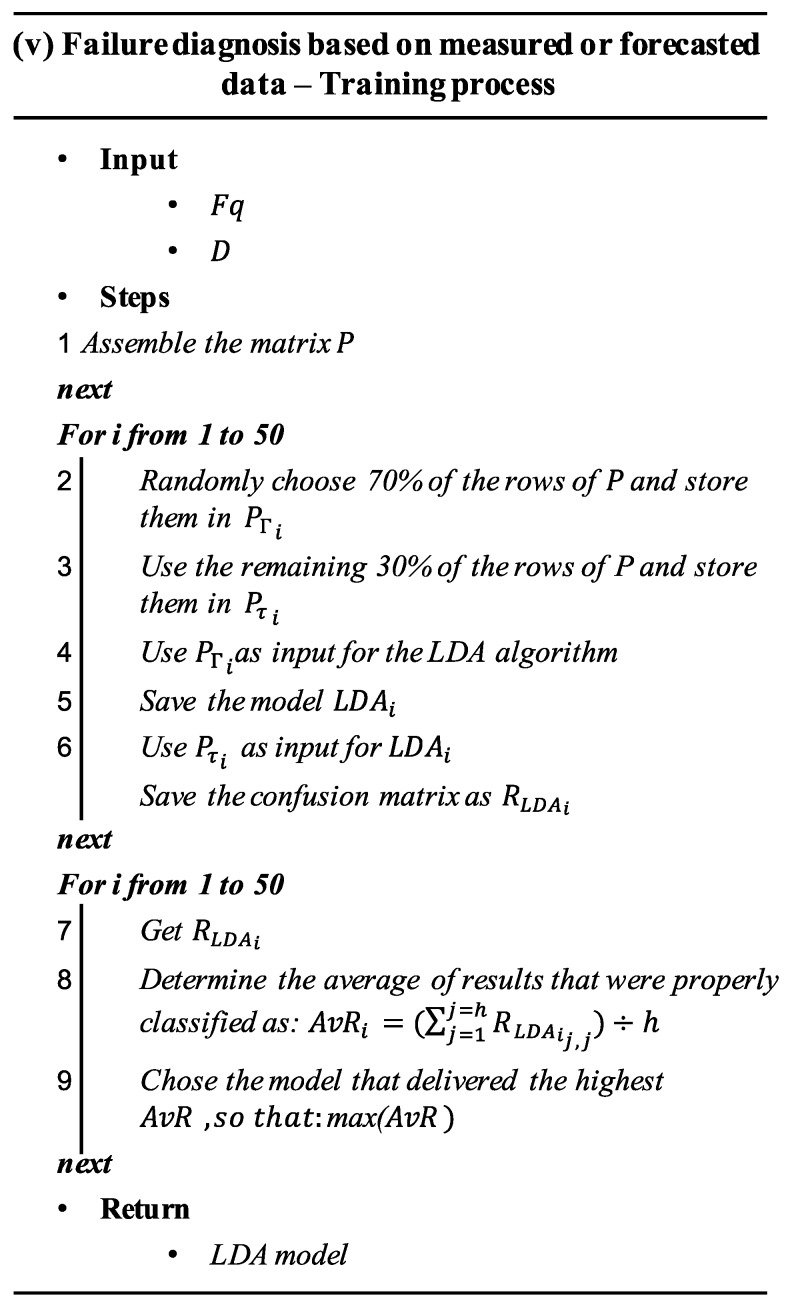
Pseudo-algorithm of the training process corresponding to step v.

Failure diagnosis makes use of a classification model that facilitates discrimination of failure modes based on $Fq^{\prime }$ (or Fq) and $D^{\prime }$ (or D) datasets. To derive a classification model, data of Fq and D, measured when the system performance was no longer acceptable (during the critical threshold of faults), were collected in a predictor matrix, P=[Fq D| F]. This matrix also included the class vector *F*, which denotes the occurring failure mode or fault-free ($F_{f}$) system’s state of each data sample in the matrix. P matrix was generated based on different ‘Failure modes’ $\left(F_{r}\right)$ at their corresponding thresholds. A detailed description of the training process was described in the algorithm (iv) presented in Figure 6.

**Figure 7:**
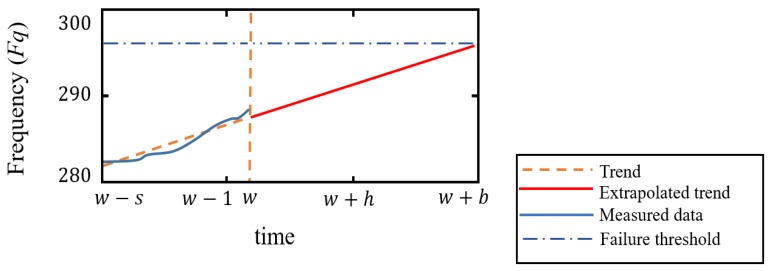
Forecasting notation.

Once the training process was completed and a classification model is available, a predictor vector for each step w was generated in order to determine (by the use of the already derived classification model) whether the observed data belongs to the Fault-free $\left(F_{f}\right)$ set or it indicates a forming fault. Unlike the training process, where *P* is a matrix composed by multiple rows, in the classification process $P_{w}$ is a vector that is denoted as:

(10) $$P_{w}=[Fq_{1_{w}},Fq_{2_{w}},\ldots ,Fq_{l_{w}}\;|\;D_{1_{w}},D_{2_{w}},\ldots ,D_{l_{w}}]$$

where l is the total number of SOMs. For conducting failure prognosis, forecasted data are arranged into a predictor vector ${\hat{P}}^{w+h}$, which is defined as:

(11) $${\hat{P}}^{w+h}=[{{\mathrm{Fq}}^{\prime }}_{1_{w+h}},{{\mathrm{Fq}}^{\prime }}_{2_{w+h}},\ldots ,{{\mathrm{Fq}}^{\prime }}_{1_{w+h}}\;|\;{D^{\prime }}_{1_{w+h}},{D^{\prime }}_{2_{w+h}},\ldots ,{D^{\prime }}_{l_{w+h}}]$$

where w+h represents one of the forecasted time periods $t^{\prime }$ included in the forecasting horizon (between w and w+b). Values w+h are used for prediction, so that, w=O can be determined. w=O denotes the step in which a particular fault becomes detectable (see Figure 7).

All forecasted observations from w+1 to w+b are delivered to the classification model in order to determine the forming ‘Failure mode’ and TTF. In this article, TTF is interpreted as the time that remains before a fault reaches its critical threshold, and it is mathematically noted as *c*. The instant of time t+c denotes the first forecasted fault progression w+h that is no longer classified as fault-free ($F_{f}$). TTF is represented in ‘fault progress steps’ (w), as every w is equivalent to L time instants.

**Figure 8:**
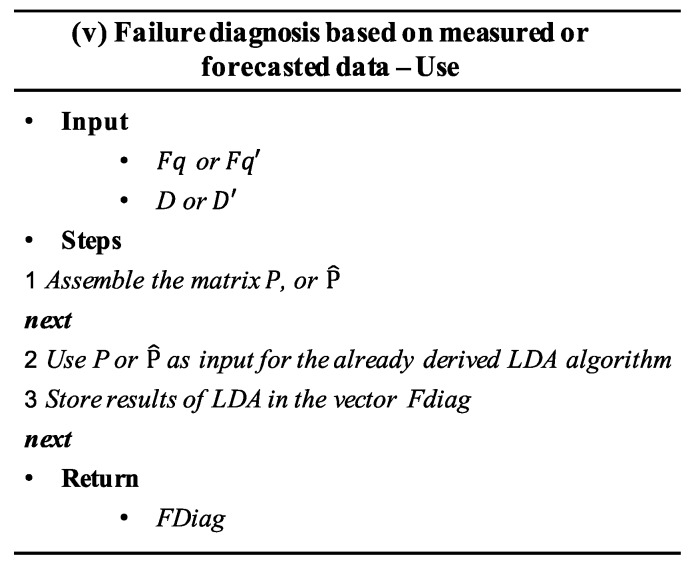
Pseudo-algorithm for the diagnosis process in step v.

The pseudo algorithm (v) of classification is shown in Figure 8. In this pseudo-algorithm *FDiag* denotes the vector that stores the results of a classification process completed during the use of the model.

Once the results from the failure diagnosis and failure prognosis processes are obtained, they should be delivered to the system operators, who shall take decisions about the most suitable actions to be performed.

## 5. Validation of the Proposed Method

To validate our hypothesis (i.e., variations on the frequency and duration of SOMs provide sufficient means for conducting failure prognosis in running time) we conducted an experiment on a small-scale cyber-physical greenhouse testbed. Two aspects were evaluated:

(1) Fq and D provide sufficient information for accurately tracing the fault evolution process.
(2) Fq and D contain sufficient information for estimating the TTF and diagnosing the forming failure mode.

The above-mentioned hypothesis, as well as the proposed prognosis method were evaluated through an experimental approach that aims to provide real-life conditions for analyzing fault manifestations. For this purpose, a testbed of a small-scale cyber-physical greenhouse was designed and built. The testbed enables to systematically induce and reproduce system failures for experimental purposes. A description of the setup of the testbed and the conducted experiments is presented below.

### 5.1. Description of the Setup of the Testbed

The instrumented small-scale cyber-physical testbed is a scaled prototype that emulates real conditions of a fully operational greenhouse system. Greenhouse operation requires controlling several interrelated variables (e.g., temperature and soil humidity, among others), which are sensitive to environmental changes. Together with a set of tasks to be accomplished by the system (e.g., irrigation, maintaining temperature and humidity), these environmental changes require self-tuning capabilities, which are supposed to provide an optimal environment for plant growth, despite varying climate conditions.

Based on [30], the instrumented testbed was composed of three main architectural layers, namely:

- Controlled core area (CCA).
- Extended field of application (EFA).
- Cross domain networking (CDN).

**Figure 9:**
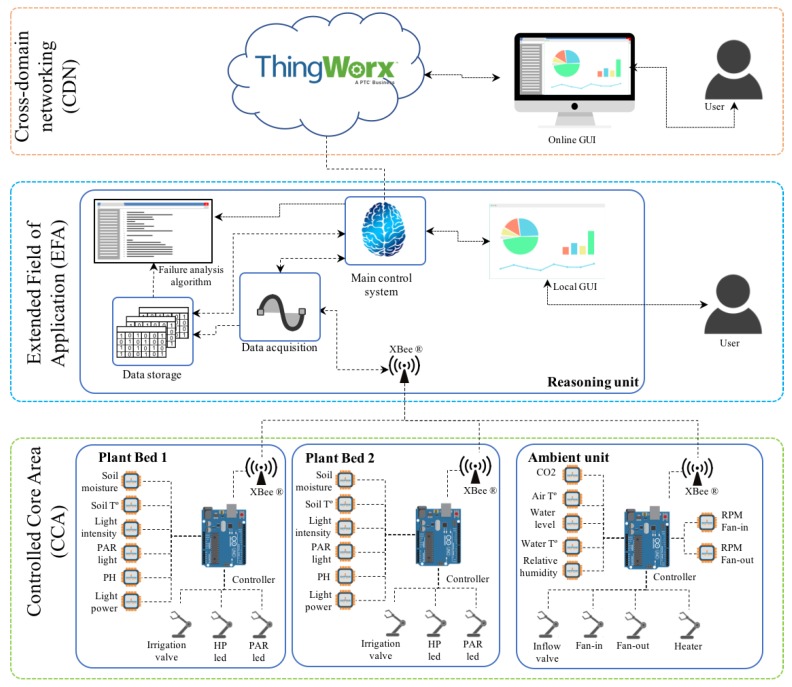
Architecture of the testbed.

These three layers describe a hierarchical system (as shown in Figure 9) where, CCA is composed by embedded systems that accomplish simple control tasks (e.g., irrigation, lighting, temperature control, humidity control and CO_2_ control). The EFA coordinates the embedded systems and takes high-level decisions (e.g., system performance optimization and the execution of the proposed failure analysis algorithm). Then, CDN enables the interaction with external systems.

**Figure 10:**
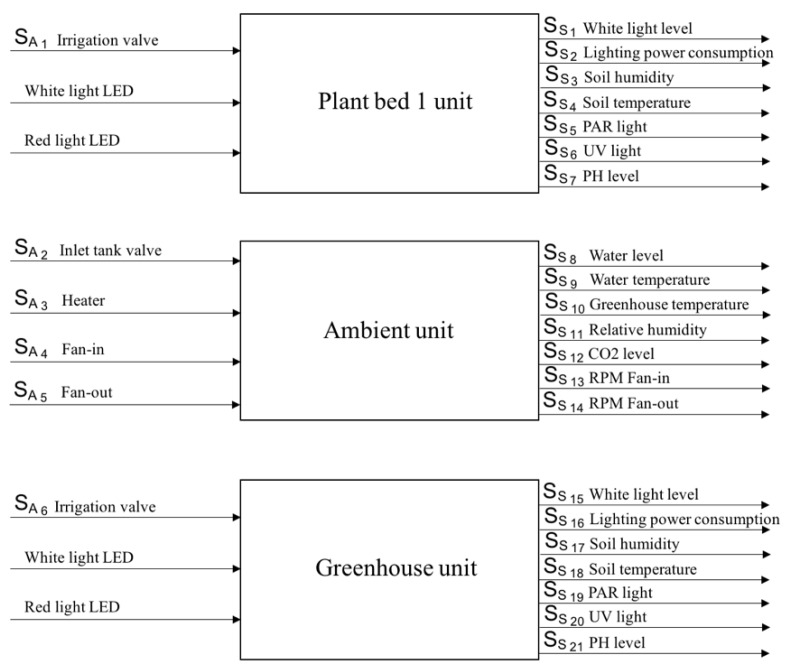
Description of the system units.

The CCA is the most relevant part of the greenhouse, considering the experimentation purposes. It controls the operation of the system and it compensates failures by self-tuning the control parameter in order to safeguard system stability. At the CCA, the system is equipped with a set of system sensors and a set of system actuators ($S_{A_{i}}$) that are interconnected through three feedback controllers (as shown in Figure 10): two for controlling plant bed conditions and one for controlling environmental conditions in the greenhouse cabin.

Actuator signals $S_{A}$ are binary signals (0 or 1), where 0 represents the inactive state of a particular component and 1 is the active state [28]. Table 1 presents a description of the actuators installed in the greenhouse testbed. Likewise, Table 2 presents the list of SOMs that can be observed during the operation of the greenhouse.

**Figure 11:**
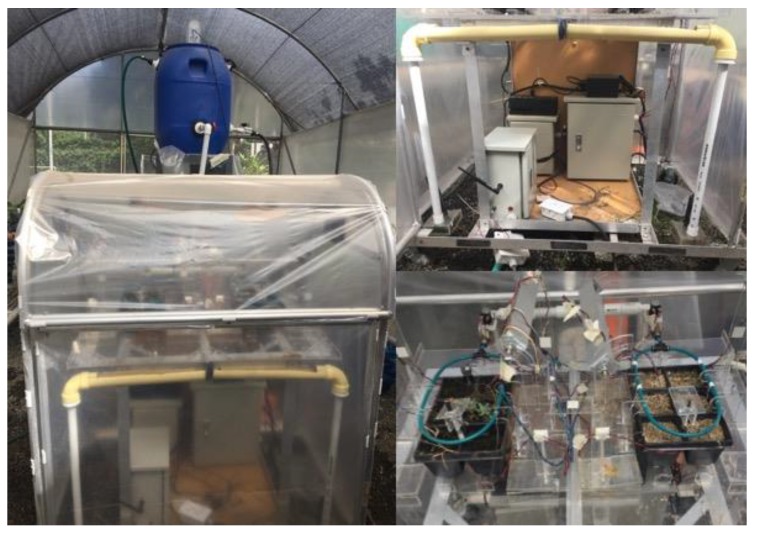
Instrumented cyber-physical greenhouse testbed.

The obtained data was processed with Matlab^®^ at the EFA layer. It enabled extracting and computing the frequency and duration of SOMs. Thingworx IoT^®^ platform was also implemented to provide a user interface that enabled collecting data from different sources, remote monitoring and modifying system set points. The instrumented cyber-physical greenhouse is shown in Figure 11.

### 5.2. Description of the Experiment

In order to validate the proposed failure forecasting method, a failure injection strategy that allows systematic induction (one at time) of different failure modes (or fault types) into the testbed was implemented. The three main failure modes experimentally induced, were:

- ’Tank leak’ $F_{1}$: a drain valve was installed on one of the walls of the water reservoir. The valve was placed close to the bottom of the reservoir and below the inlet and the outlet valves.
- ‘Irrigation pipe blocked’ $F_{2}$: The irrigation hole located next to the ‘Soil moisture sensor’ of plant bed 2 was obstructed with Teflon tape. The manipulated variable was the flowrate of irrigation (‘Electro valve plant bed 2′ $S_{A_{6}}$).
- ‘Irregular fan operation’ $F_{3}$: A resistance that reduces the electrical current that feeds the fan was installed, in order to modify the regular speed of rotation of the inlet air fan. The manipulated variable was the RPM of the fan (‘Fan In’$S_{A_{4}}$).

**Figure 12:**
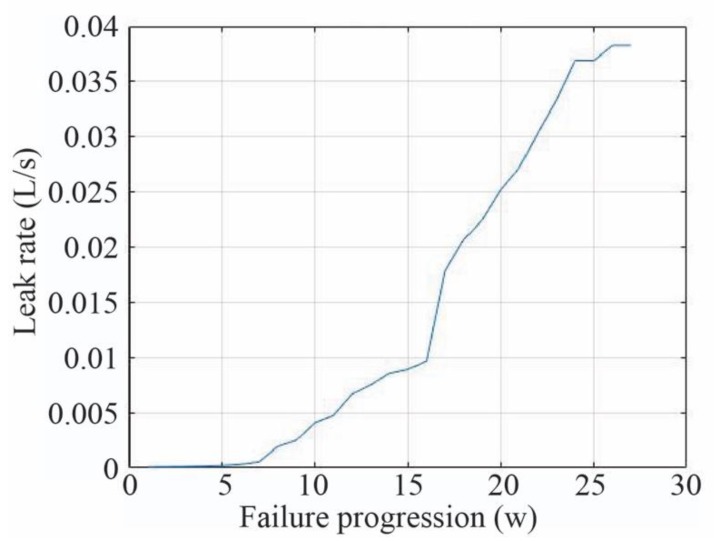
Evolution of the greenhouse ‘Tank leak’ failure mode (F_1).

In the case of $F_{1}$, ‘Tank leak’ $F_{1}$ was incrementally increased with 27 levels of fault progression. It started with a leak rate of 0.000085 L/s and ended with a leak rate of 0.038 L/s at its critical threshold, when the system failure occurs. $F_{2}$ and $F_{3}$ were only injected at their critical threshold. Figure 12 depicts the progress of the fault. Fifteen different experiments, in which the same fault progression process was injected, were conducted. These experiments were performed in different days, which presented variations in the ambient temperature, light intensity, air humidity and water temperature.

### 5.3. Evaluation of the Trend Consistency and Discriminant Power

Our hypothesis on the proposed failure prognosis method is that frequency and duration of SOMs can enable to reliably monitor the fault forming process (or degradation process), as well as determining the occurring failure mode. In this evaluation process, we analyzed:

(1) The consistency of trends (of Fq and D), when produced by the same ‘Failure mode’ $F_{r}$;
(2) The discriminant power for distinguishing different ‘Failure modes’ ($F_{r}$).

It can be argued that, if observed trends from different datasets but the same ‘Failure mode’, $F_{r}$, have strong similarity, it confirms that $F_{r}$ consistently modifies the behavior of the system. Likewise, if the observed trends triggered by $F_{r}$ are different from those triggered by a different failure mode, it can be inferred that Fq and D have discriminant power.

Statistical tests were implemented for evaluating the degree of similarity between the trends presented by the frequency and duration of SOMs as an effect of failures. In order to carry out this analysis, the trends of Fq and D were extracted through a Savitzky–Golay filter. It allows filtering out the effect of external factors such as variations on the use and operation conditions. Then, the variation of the SOM frequency ΔFq and SOM duration ΔD, as the fault progresses was evaluated (i.e., how much the SOM frequency of occurrence (Fq) and the average SOM duration D changes as the fault progresses), considering that:

(12) $$\Delta Fq=Fq_{w=n}-Fq_{w=1}$$

(13) $$\Delta D=D_{w=n}-D_{w=1}$$

In both equations, n denotes the last failure step progress considered in the analysis.

Due to the fact that trends are better characterized by non-parametric distribution, Kruskal–Wallis [31] (with a Bonferroni correction) was applied for comparing their similarity. This analysis was conducted for ΔFq and ΔD separately, thus, two null hypotheses $H_{0}$ were formulated:

- The observed ΔFq, collected from different experiments and different failure modes, belongs to the same distribution. That is, they are equal.
- The observed ΔD, collected from different experiments and different failure mode, belongs to the same distribution.

p-value was used for rejecting the hypotheses. The threshold considered is p=0.05, where the rejection is determined by p≤0.05. A pairwise comparison (of $\Delta Fq_{\varsigma_{d}}^{F_{f}}$ with $\Delta Fq_{\varsigma_{d}}^{F_{r}}$ and $\Delta D_{\varsigma_{d}}^{F_{f}}$ with $\Delta D_{\varsigma_{d}}^{F_{r}}$) was conducted. For this, the SOM is represented by $\varsigma_{d}\;F_{f}$ denotes the fault-free case and $F_{r}$ denotes the failure Mode (which for this case is ‘Tank leak’ $F_{1}$).

The observed results revealed that the fault-free ($F_{f}$) cases did not show any particular trend as the effect of the fault-free behavior for none of the 15 experiments. However, clear trends were observed in data corresponding to $F_{1}$, more specifically, in the cases of SOMs $\varsigma_{11}$, $\varsigma_{13}$, $\varsigma_{15}$, $\varsigma_{41}$, $\varsigma_{45}$ and $\varsigma_{47}$ for the Fq indicator. Similar trends were observed in all analyzed SOMs when analyzing D for $F_{1}$, except by $\varsigma_{11}$, $\varsigma_{13}$ and $\varsigma_{43}$.

**Figure 13:**
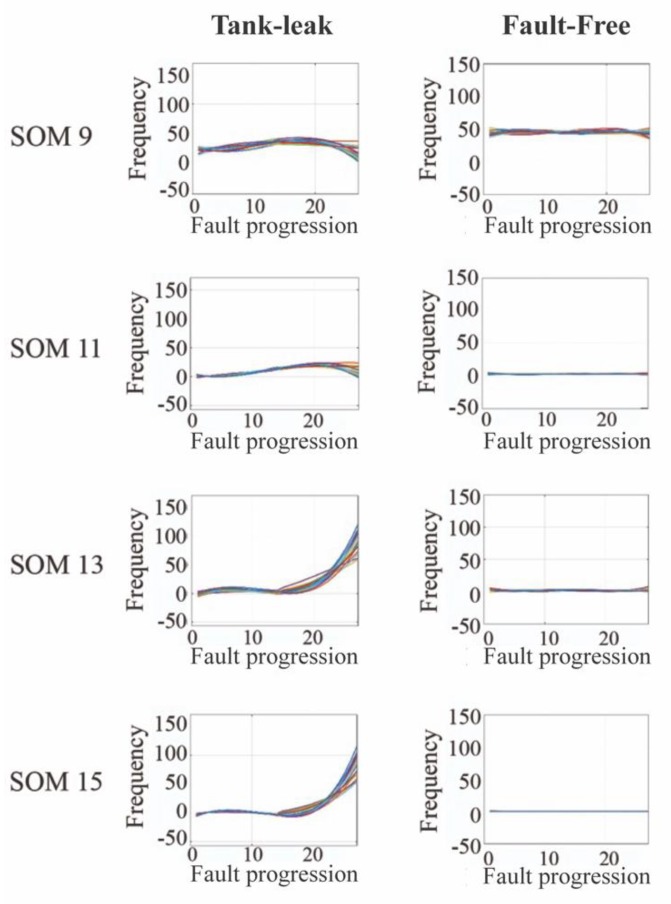
Comparison between the variations observed in the frequency of system operation mode (SOM), when subjected to a tank-leak, versus the variation observed in the fault-free state.

Results presented in Table 3 indicate upward or downward trends in the failed cases. These trends differed from the steady behavior observed in the fault-free cases (see Figure 13). Eventually caused by the emerged faults, the observed trends were triggered by the system control, which changed the frequency and duration of SOMs with the aim of compensating for the effect of faults. Moreover, the observed trends in the ‘Tank leak’ $F_{1}$ experiments present trajectories that were consistent with each other. It implies that the external factors caused by real-life environmental conditions did not have a significant effect on the observed trends.

The variance of *Fq* and *D* were also analyzed for the different sets of curves corresponding to the same SOM and the same failure mode. The obtained results are summarized in the Table 4. It can be observed that variance results were below 0.09, indicating that observed trends from different curves and the same failure mode were consistent. It suggests that different failure events corresponding to the same failure mode manifested similarly through *Fq* and *D*, enabling their use for failure detection. This situation, along with the difference observed when comparing the trends from the fault-free case and those from tank leak, implied that frequency and duration of SOMs could be used as indicators of fault evolution.

### 5.4. Evaluation of the Frequency and Duration of SOMs as Indicators of Fault Progression

In this analysis the same data were used that was previously utilized for evaluating the main assumptions of the failure prognosis concept. For this purpose, Fq and D were extracted from the 27 fault progressions (w) described in Section 4.2. The evaluation of the capabilities of Fq and D for tracing fault progress, requires building a classification model by following the process described in step (iv) of the proposed method. A linear discriminant analysis was conducted in order to derive this model, which used as input the collected data at the critical threshold of the ‘Tank leak’ ($F_{1}$) at w=27 (i.e., when the leak rate reaches 0.038 L/s). A dataset consisting of 70 samples was used for training and testing purposes. It included:

- Twenty data samples corresponding to SOM frequency (Fq) and SOM duration (*D*) gathered during the fault-free $F_{f}$ operation of the testbed;
- Twenty data samples corresponding to ‘Irrigation pipe blocked’ ($F_{2}$) representing a complete blockage of one of the irrigation pipes at plant bed 2;
- Nine data samples corresponding to ‘Irregular fan operation’ ($F_{3}$).

Fifty random datasets were generated by partitioning the total dataset. From each dataset 70% of data were used for training the failure classifier. The remaining 30% of data, were used for testing. The selection of the best model was performed by maximizing the accuracy of the classification (successful classification rate) for each class, so that, none of the failure modes would present a low classification accuracy. Samples used for the analysis presented in Section 4.3 were included for deriving the classification model. The results obtained through the test set are summarized in Table 5, which is the confusion matrix of the results for ‘Tank leak’ $\left(F_{1}\right)$*,* ‘Irrigation pipe blocked’$\left(F_{2}\right)$ and ‘Irregular fan operation’ $(F_{3}$). The confusion matrix of the selected model did not present any false-negative nor false-positive result for any of the analyzed failure modes. All data samples considered during the test were successfully classified.

**Figure 14:**
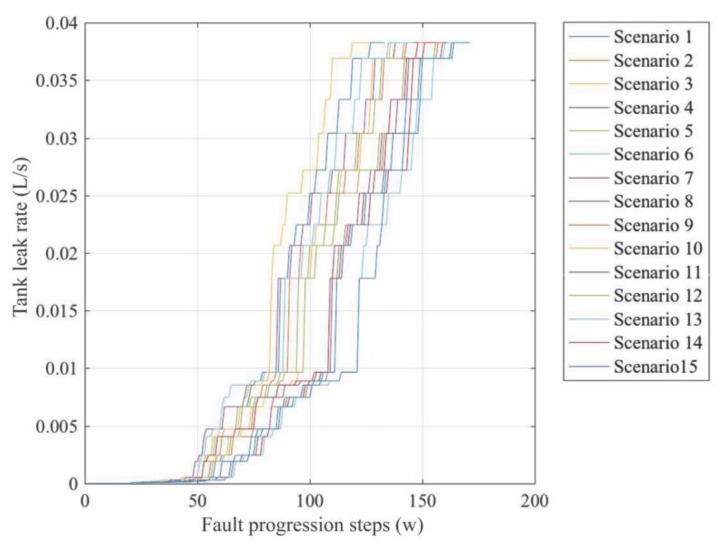
Fault progression processes.

Once the model was available (delivered and tested) Fq and D were computed (15 data samples per fault progression level w were considered), in order to use the derived model for training the algorithm through failure diagnosis. This analysis considered 15 different scenarios of fault evolution. For this purpose, data belonging to the dataset of every w step was randomly selected and sequentially arranged in 15 different cases (see Figure 14). Unlike the analysis of assumptions (Section 4.3) where the fault size at w was different than the fault size at w−1 and w+1, the current analysis can present the same fault size during several consecutive *w* steps. The first 20 fault progression steps correspond to fault-free ($F_{f}$) system operation.

The analysis conducted aimed to determine to what extent the observed SOMs’ frequency (Fq) and duration D indicators managed to predict the occurring ‘Failure Mode’ $F_{r}$ at different levels of the degradation process. For this purpose, the Fq and D of every fault size were used as input for the derived classification model. This process was conducted with the aim to determine at which fault progression step (w) failures started to be diagnosed correctly.

**Figure 15:**
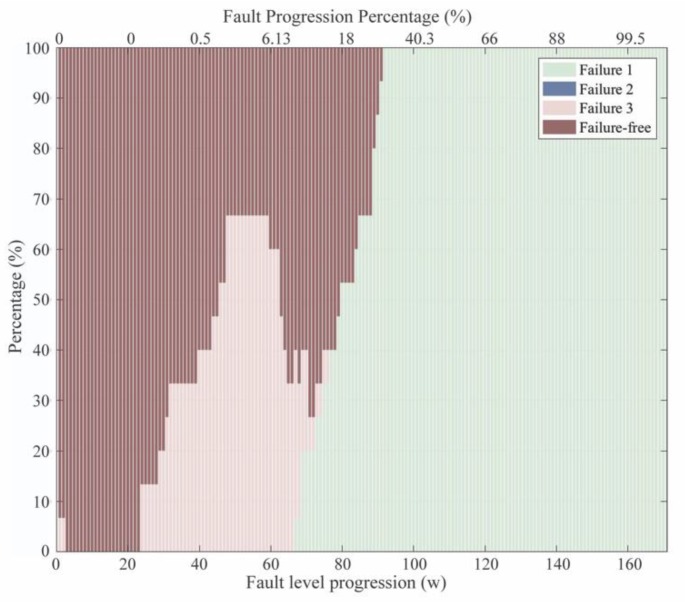
Evolution of failure prediction for failure-leak $\left(F_{1}\right)$.

The results of the investigation are graphically presented in a bar-plot in Figure 15. Every single bar represents the distribution of the predicted failures for the analyzed scenarios (15 scenarios) in every step w. A code of colors indicates the predicted failure modes, as it is shown in the legend. The distribution of the analyzed experiments is presented in the y-axis, while the x-axis represents both, the fault progression (w) and the fault progression percentage (FPP). FPP expresses the advancement of the fault formation and it was estimated by considering the progression step w=172 as the fault size = 100%.

It can be observed that ‘Tank leak’ *(*$F_{1})$ was recognized and properly diagnosed from step w=67, when the first scenarios reached a leak rate of 0.0089 L/s (FPP = 2.34%). However, it was only at the step w=92 when all analyzed scenarios reached this leak rate. From that progression step, failure diagnosis was consistent and remained stable until reaching the critical threshold of the fault. It could be observed, that a number of cases were misclassified as ‘Irregular fan operation’ $\left(F_{3}\right)$ between w=24 (FPP = 0.23%) and w=76 (FPP = 15.75%), while real failure mode was a tank leak. There are even some cases at w=1 and w=2 where 7% of the analyzed samples were misclassified as $F_{3}$, while it was actually fault-free ($F_{f}$). Between w=48 (FPP = 0.84%) and w=59 (FPP = 6%) the number of misclassified scenarios as $F_{3}$ reached its maximum at 67% of the analyzed cases. This phenomenon demonstrated that there was a short period in the early fault stages in which fault characteristics were misclassified for another failure mode, but as the fault progressed the correct diagnosis of $F_{1}$ appeared.

This is interesting to analyze as it kept occurring during 43 consecutive fault progression steps. There can be two main hypothetical answers to the observed misclassification: (i) the low number of data samples corresponding to $F_{3}$ considered during the training of the classification model or (ii) the lack of a characteristic manifestation of $F_{3}$ through Fq and D indicators.

Another explanation is that the inlet fan $(S_{A}{}_{4}$= ‘Fan-in’) seeks to control the ambient temperature and CO_2_ concentration levels in the greenhouse. However, there are no further control actions that involve the components affected by the failure mode $\left(F_{3}\right).$ The ‘Irregular fan operation’ $\left(F_{3}\right)$ was observable through variations of the intensity of operation of the actuator $\left(S_{A_{4}}\right)$. However, frequency *(*Fq) and duration *(*D) indicators were only based on on/off states of $\left(S_{A_{4}}\right)$. This made the failure diagnosis method incapable to distinguish between the fault-free ($F_{f})$ case and $F_{3}$.

The misclassification problems cannot be linked to external factors, as they had consistent behavior across the analyzed scenarios. However, the current concept of Fq and D of SOM has limitations when control actions are increasing or decreasing the intensity of the actuator’s operation (i.e., when the actuator is analogous). Intensity of actuator’s operation is a factor that should be considered in the definition of SOMs. It describes a component operation mode that keeps the stability property by modifying the power of operation of the actuator, instead of just activating or deactivating it. Although, this new dimension is a very interesting aspect, it was out of the scope of the present article.

### 5.5. Evaluation of the Utilization of Frequency and Duration of SOMs for Prognosis Purposes

**Figure 16:**
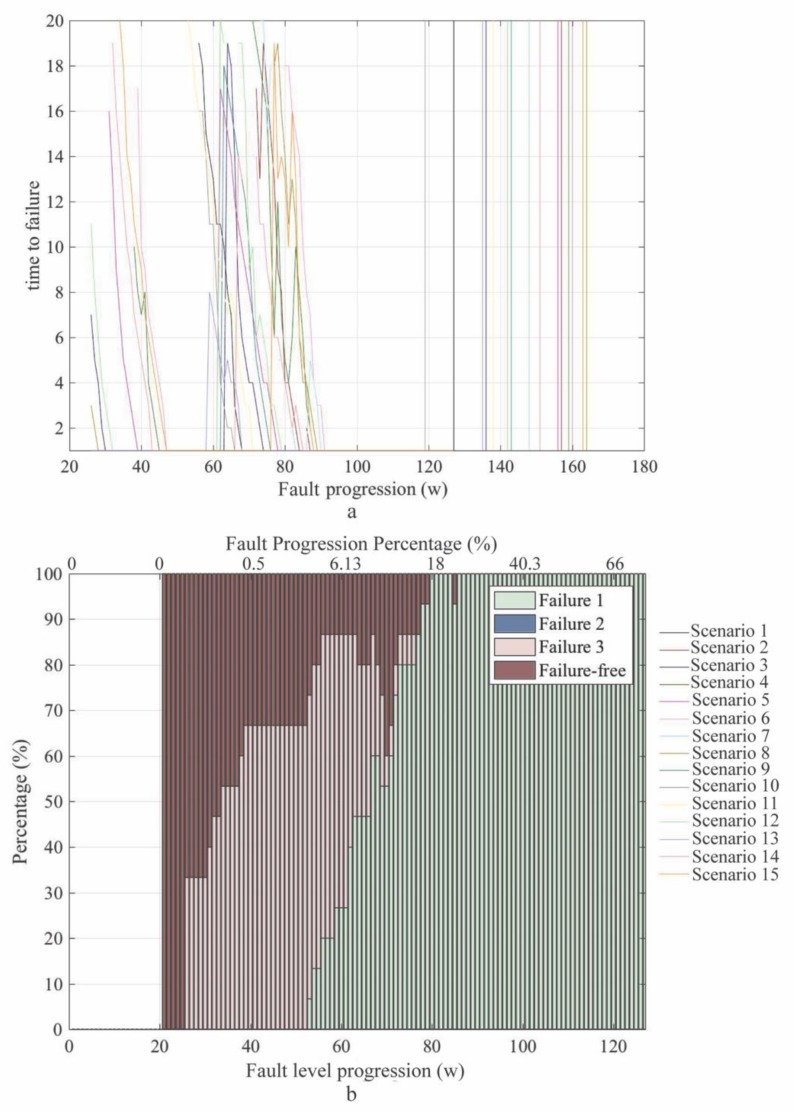
Failure forecasting for tank leak; (**a**) time-to-failure (TTF) of the failure mode and (**b**) forecasting of the failure mode.

This section evaluates the implementation of failure prognosis through forecasted data of Fq and D. For this purpose, the classification model previously presented in this article was fed with forecasted data. In order to derive the prognosis model, the 15 scenarios presented in Figure 14 were included in this analysis. Moving windows with s = 20 steps and a forecasting horizon of b=20 were also considered. Results obtained from the prognosis are presented in Figure 16. This figure presents the evolution of the TTF estimation (Figure 16a) and the predicted failure mode (Figure 16b).

In Figure 16a, the x-axis represents the fault progression steps w, while the y-axis is the estimated TTF. Note that values on the y-axis are in the range of 0 and 20, considering that b=20. The limits of the x-axis ranges between 20 and 180, as there were no failure predictions on the forecasting horizon before w=20. The results of different simulation scenarios were combined into a single diagram, where each individual curve represents one of the 15 experiments. The vertical lines on the right side of the plot are the fault’s critical threshold for every experiment. The color of the threshold line is the same as the color of the curve corresponding to its corresponding experiment.

The graph depicted in Figure 16b shows a similar pattern to the one observed in Figure 15. However, the use of forecasted Fq and D indicators as input for the classification model facilitates the recognition of the occurring failure mode earlier (around 15 progressions *w* before). Two sets of curves could be identified in the TTF plot at Figure 16a. The first set was between steps w=27 (fault size = 0.27%) and w=48 (fault size = 0.84%) and the second one was between steps w=60 (fault size = 6.13%) and w=98 (fault size = 39%). The first set predicted a TTF ≈67 fault progression steps for scenarios 3, 4, 5, 6, 8, 9, 12, 13 and 15 (see Figure 14). Although these scenarios were misclassified as $F_{3}$ in the first set, their prediction gradually moved to $F_{1}$ in the second set, as the fault progressed (as shown in Figure 16b). The reason is the estimated TTF changed gradually, as the actual step w approached the critical threshold (represented by the vertical lines that can be observed in Figure 16a).

Based on the presented results, it can be observed that the predicted TTF was shorter than the actual TTF. The failure prognosis analysis delivered an average TTF=84 for TTFs estimated for the steps between w=73 and w=98. However, the actual average fault threshold was at step w=146 as failures from the analyzed scenarios occurred between w=127 and w=171.

In all analyzed cases, the observed trends of the SOMs’ frequency (Fq) and duration (D) indicators provided relevant information that enabled failure prognosis. The observed results demonstrated that Fq and D could be used to predict the type of the forming fault from early stages. However, the obtained TTF results were somewhat inaccurate. It is argued that, obtaining premature TTF estimations is preferable to avoid failure occurrence, rather than not having information at all about forthcoming system failures. However, this premature prediction may cause the replacements of components that can still be used longer. These results may affect the trade-off between safe operation and optimized maintenance, but, still, it is important to have this information for decision-making processes.

## 6. Discussion

The analysis conducted aimed to evaluate the hypotheses that underpin the proposed health management and failure prognosis method. The obtained results demonstrated that degradation and fault evolution was manifested through variations of the frequency and duration of SOMs, which were influenced by the effect of the compensatory actions, carried out by self-tuning systems. SOM frequency and duration allows monitoring fault progress based on data derived from control signals. This last characteristic is relevant, as it enables considering variations of system behavior and environmental factors that can affect the lifetime of the system.

Frequency and duration of SOMs can be considered fault indicators. They allow studying the system degradation process. In the conducted experiments, trends of SOM frequency and duration showed strong similarity for different events corresponding to the same failure mode. They also showed significant differences between trends from different failure modes. A combined analysis of the trends of SOM frequency and duration showed that they had high discriminant power enabling failure diagnosis.

The discriminant power of Fq and D is very relevant in the context of CPSs. The reason is, the multiple potential applications these types of system can present makes it needed identifying the parameters that reveal fault symptoms in every single application. However, considering that Fq and D are supported on SOM activation (which occur in all the CPS applications), it can be said these indicators can be extrapolated to other types of small-scale CPSs, different from small-scale cyber-physical greenhouses. Moreover, they can be used for multiple failure modes, making the automation of the failure diagnosis process easier in small-scale CPSs.

The availability of a generic indicator for failure analysis represents a step forward towards the advent of further generations of CPSs. The reason is, the adaptable and evolvable behavior these types of systems will present strengthens the need of having indicators that work properly despite changes on system configuration, and system topology.

The evaluation of the implementation of Fq and D for failure prognosis showed that it was possible to analyze the future system reliability by forecasting the trends of the SOM frequency and duration. It allowed estimating the TTF of the system as well as determining the forming failure mode. Nevertheless, the TTF was somewhat inaccurate. Further studies are required around this subject, in order to provide a more accurate estimation of TTF using compensatory methods. Although it is always desired knowing in advance the time remaining before failure occurs, inaccurate estimations may affect negatively the cost-effective implementation of maintenance strategies.

Results concerning the discriminant power of the indicators, as well as their potentials for prognosis, were positive. They suggest that SOM frequency and duration are suitable predictors for diagnosing progressive faults. Nevertheless, it can prevent the detection of failures that are manifested through transient faults. Transient faults manifest intermittently, i.e., they are not manifested through long-term trends and therefore they can be mistaken as noise effect. This particular aspect requires further study as the analysis here presented is based on the historic manifestations of failures.

Finally, the proposed method is addressed to small-scale CPSs. Their application in large-scale CPSs may present problems, due to large number of possible COM combinations that a CPS composed by many components have. Further studies should be conducted in order to determine the maximum CPS size in which this method is still valid.

## 7. Conclusions

In this article, a failure diagnosis and prognosis method for small-scale, self-tuning cyber-physical systems was proposed. Self-tuning cyber-physical systems are characterized by their capability of modifying their system settings autonomously with regards to adapting themselves to the varying working and environmental conditions. This characteristic is desirable, as it provides these types of systems with fault tolerance capabilities and it fosters a more efficient system operation. Nevertheless, it can also mask fault symptoms hampering a timely fault detection and diagnosis.

The proposed method is underpinned on the varying frequency (Fq) and duration (D) of system operation modes. These two parameters present gradual changes as the effect of the fault compensation actions exerted by the system with regards to keep system stability. The principle underpinning this research is that analyzing the evolution of the frequency and duration of SOMs as the fault progresses, it is possible to overcome the failure masking effect that present self-tuning CPSs, as well as providing means for failure prognosis. This health management and prognosis method used Fq and D as descriptors for a LDA-based classification model that aims to discriminate the occurring failure mode, while exponential smoothing was used for predicting the forthcoming values of Fq and D, in order to estimate the system’s time to failure (TTF).

The assumptions underpinning the method, as well as its capability for failure prognosis were tested through an experimental approach where a small-scale, self-tuning cyber-physical greenhouse was instrumented in order to systematically inject different failure modes. It was found that, changes in the frequency and duration of system operation modes caused by a self-tuning system have a discriminative power in terms of failure classification, enabling failure diagnosis. Likewise, these changes show a strong correlation with the trends of system degradation, making it possible the monitoring of the fault progress with regards to failure prognosis. One of the main advantages of this method is that Fq and D can be used as fault indicators for different failure modes, enabling its use in multiple types of small-scale cyber-physical systems.

Results obtained demonstrated that the proposed method manage to discriminate and to predict the forming failure mode. Nevertheless, some aspects still require further study:

(1) Firstly, a proper validation of the frequency and duration of SOMs for failure diagnosis and prognosis should be performed. In the present work, only a case study based on an instrumented testbed was conducted. However, a representative set of several and heterogeneous types of self-tuning CPSs should be considered with the aim to test the limitations of the proposed concept.
(2) Secondly, the analysis of variations of actuators intensity is also required. So far, variations on frequency and duration of SOMs as means of self-tuning have been analyzed. However, variations on actuators’ operation intensity can also provide relevant information about the fault forming process.
(3) Thirdly, more fault forming patterns should be evaluated to determine their effect over the frequency and duration of SOMs. Due to their heterogeneous nature, faults can present multiple variations on their associated degradation process that can affect the analyzed long-term trends. For instance, faults with non-linear progression trends may influence the changes of frequency and duration of SOMs. It is argued that they cause sudden upward or downward trends that could affect the estimations of TTF, as well as the prediction of the forming failure mode.

The above-mentioned factors can be addressed in further investigations. Nevertheless, the present work is a step towards the understanding the potential of the proposed failure diagnosis and prognosis concept.

## Figures and Tables

**Table 1:** System components description.

System Component	Variable	Description	Domain/Set-Point
Electro valve Plant bed 1	$S_{A_{1}}$	Irrigation valve of Plant bed 1	$E_{S_{A_{1}}}=\left\{ValveClose,ValveOpen\right\}$
Electro valve water reservoir	$S_{A_{2}}$	Inlet tank valve	$E_{S_{A_{2}}}=\left\{ValveClose,ValveOpen\right\}$
Heater	$S_{A_{3}}$	Water resistance for the heater	$E_{S_{A_{3}}}=\left\{ResistanceOff,\;ResistanceOn\right\}$
Fan-in	$S_{A_{4}}$	Fan-in of the central unit	$E_{S_{A_{4}}}=\left\{\mathrm{Fan-inOff},\;\mathrm{Fan-inOn}\right\}$
Fan-out	$S_{A_{5}}$	Fan-out of the central unit	$E_{S_{A_{5}}}=\{\mathrm{Fan-inOff},\;\mathrm{Fan-inOn}\}$
Electro valve Plant bed 2	$S_{A_{6}}$	Irrigation valve of Plant bed 2	$E_{S_{A_{6}}}=\left\{ValveClose,ValveOpen\right\}$

**Table 2:** Occurring system operation modes.

SOM	$S_{A_{1}}$	$S_{A_{2}}$	$S_{A_{3}}$	$S_{A_{4}}$	$S_{A_{5}}$	$S_{A_{6}}$
9	off	off	off	on	off	off
11	off	on	off	on	off	off
12	off	on	on	on	off	off
13	off	off	on	on	off	off
15	off	on	on	on	off	off
33	off	off	off	off	off	on
41	off	off	off	on	off	on
43	off	on	off	on	off	on
45	off	off	on	on	off	on
47	off	on	on	on	off	on

**Table 3:** Results of the statistical test for SOM frequency and SOM duration.

	*p*-Value							
	SOM 9	SOM 11	SOM 13	SOM 15	SOM 41	SOM 43	SOM 45	SOM 47
**Frequency**	0.065	0.017	3.07 × 10^−6^	3.05 × 10^−6^	6.26 × 10^−5^	0.15	3.07 × 10^−6^	6.12 × 10^−7^
**Duration**	3.07 × 10^−6^	0.98	0.95	3.73 × 10^−6^	5.25 × 10^−5^	0.78	0.00097	6.12 × 10^−7^

**Table 4:** Variance per SOM in the greenhouse’s case.

	Tank Leak’s Variance—Greenhouse Case							
	SOM 9	SOM 11	SOM 13	SOM 15	SOM 41	SOM 43	SOM 45	SOM 47
**Frequency**	0.021	0.002	0.013	8.90 × 10^−5^	0.0087	9.76 × 10^−5^	0.0004	0
**Duration**	0.086	9.87 × 10^−5^	0.055	0.0008	2.80 × 10^−6^	0.0004	0.0016	0

**Table 5:** Confusion matrix of the greenhouse’s classification model.

Failure Mode	*F*_1_	*F*_2_	*F*_3_	Failure-Free
***F*_1_**	6	0	0	0
***F*_2_**	0	6	0	0
***F*_3_**	0	0	2	0
**Failure-free**	0	0	0	6
